# Association between FIB-4, all-cause mortality, cardiovascular mortality, and cardiovascular disease risk among diabetic individuals: NHANES 1999–2008

**DOI:** 10.3389/fcvm.2023.1172178

**Published:** 2023-09-25

**Authors:** Lihua Guan, Lei Li, Yutong Zou, Jian Zhong, Ling Qiu

**Affiliations:** ^1^ Department of Laboratory Medicine, Peking Union Medical College Hospital, Peking Union Medical College & Chinese Academy of Medical Science, Beijing, China; ^2^ State Key Laboratory of Complex Severe and Rare Diseases, Peking Union Medical College Hospital, Peking Union Medical College & Chinese Academy of Medical Science, Beijing, China

**Keywords:** FIB-4, diabetes, NHANES, all-cause mortality, cardiovascular mortality

## Abstract

**Background:**

Diabetes is prevalent worldwide and is associated with cardiovascular disease (CVD). Furthermore, due to the insulin resistance, diabetic populations are vulnerable to liver fibrosis, which increases the risk of CVD. Fibrosis-4 index (FIB-4)—a non-invasive biomarker for liver fibrosis—is crucial in predicting CVD among patients with liver diseases. However, the association between FIB-4, death, and CVD in the US diabetic population has not yet been investigated.

**Method:**

We conducted a cross-sectional study using the data from the National Health and Nutrition Examination Survey (NHANES) 1999–2008. The mortality status was obtained from the National Death Index through December 31, 2015. Participants were divided into survivor and mortality group to compare the basic characteristics. The association between FIB-4, death, and CVD was analyzed using the restricted cubic spline method and Cox proportional hazards models. In stratified analysis, Participants were stratified based on age, sex, BMI, hypertension, or eGFR respectively.

**Results:**

The participants (*N* = 3,471) were divided into survivor (*N* = 1,785) and mortality groups (*N* = 1,632), with the mortality group exhibiting significantly higher FIB-4 values. Moreover, the risk of all-cause mortality (HR 1.24; 95% CI, 1.17–1.32) and CVD mortality (HR 1.17; 95% CI, 1.04–1.31) increased with each FIB-4 SD increase after adjusting for all covariates. However, except for myocardial infarction, FIB-4 had no significant effect on the incidence of the other three CVD subtypes (congestive heart failure, coronary heart disease, and angina pectoris). In stratified analysis, we found that the effect of FIB-4 on CVD mortality was influenced by age, and FIB-4 is a risk factor for people older than 60 years (HR 1.14; 95% CI, 1.01–1.29).

**Conclusion:**

Using data from NHANES 1999–2008, FIB-4 was found to be associated with all-cause and CVD mortality in the diabetic population, and this association was significantly affected by age. However, FIB-4 only affected the incidence of myocardial infarction. Future work should investigate the association between FIB-4 and CVD in the diabetic population.

## 1. Introduction

Diabetes is caused by impaired insulin production or insulin resistance and is manifested by persistent hyperglycemia. It is regarded as one of the largest global health emergencies and imposes a substantial economic burden. Currently, approximately 537 million adults are living with diabetes, half of whom are undiagnosed. Besides, the number of diabetic patients is estimated to increase to 783 million by 2045 (1). Diabetes is closely associated with cardiovascular disease (CVD). It is a risk factor for CVD and contributes to the high incidence of CVD (2). Moreover, CVD is the leading cause of death among diabetic patients, accounting for 44%–52% (3). Therefore, it is crucial to identify the risk factors for the development of CVD in diabetic patients.

Additionally, diabetic patients are vulnerable to liver fibrosis. A cross-sectional study reported that approximately 23.8% and 15.4% of US diabetic adults had, respectively, significant liver fibrosis and advanced liver fibrosis (4). The vulnerability of liver fibrosis in diabetic patients can attribute to insulin abnormalities. Insulin plays an essential role in the normal functioning of the liver. Insulin mediates the glucose intake of the liver, and insulin resistance can lead to hepatic lipid accumulation and abnormal glucose regulation, which may eventually result in liver fibrosis. In addition to insulin resistance induced by diabetes as a risk factor of liver fibrosis, hepatitis, alcohol overconsumption and non-alcholic fatty liver disease (NAFLD) also promote the development of liver fibrosis. Among them, NAFLD is most common cause of liver fibrosis, which is also highly prevalence in diabetic patients, especially T2DM patients, accounting for 55.48%. The prevalence of advanced liver fibrosis was significantly higher in patients with both T2DM and NAFLD (17.02%) than patients with only T2DM (4.80%) (5, 6). Moreover, liver fibrosis also increases the risk of CVD (7). Therefore, diabetic patients with liver fibrosis may substantially increase the incidence of CVD.

Even though liver biopsy remains the “golden standard” of diagnosis of liver fibrosis, studies have been devoted to investigating the role of some non-invasive biomarkers in diagnosing liver fibrosis, including the Fibrosis-4 index (FIB-4), which is calculated through common parameters (age, AST, ALT, and platelet counts). European Association for the Study of the Liver (EASL) (8) and the American Association for the Study of Liver Diseases (AASLD) (9) have recommended FIB-4 to stratify liver fibrosis and rule out advanced liver fibrosis based on the high negative predictive value of it. Furthermore, studies have used FIB-4 to investigate the effect of liver fibrosis on CVD in some liver diseases, such as NAFLD (10, 11). Due to the high risk of liver fibrosis in diabetes, diabetic patients may have a relatively high risk of abnormal FIB-4 value. However, to our knowledge, there is no study utilizing FIB-4 to investigate the relationship between liver fibrosis and CVD in diabetic patients.

Therefore, the present study investigated the relationship between FIB-4, all-cause mortality, and CVD mortality among the US diabetic population in the National Health and Nutrition Examination Survey (NHANES) database, which may help to identify the predictive value of FIB-4 of CVD and its poor prognosis in the diabetic population.

## 2. Method

### 2.1. Study design and population

NHANES is a cross-sectional survey reflecting the health and nutritional status of the non-institutional population in the US since the 1960s. It has evolved into a continuous survey program since 1999 by recruiting approximately 5,000 people each year. It was conducted by the National Center for Health Statistics (NCHS) of the Centers for Disease Control and Prevention (CDC), and each participant signed a written informed consent (12, 13). The detailed information analyzed in this study can be obtained from the NHANES web (https://www.cdc.gov/nchs/nhanes/index.htm; viewed on January 15, 2023).

**Figure 1:**
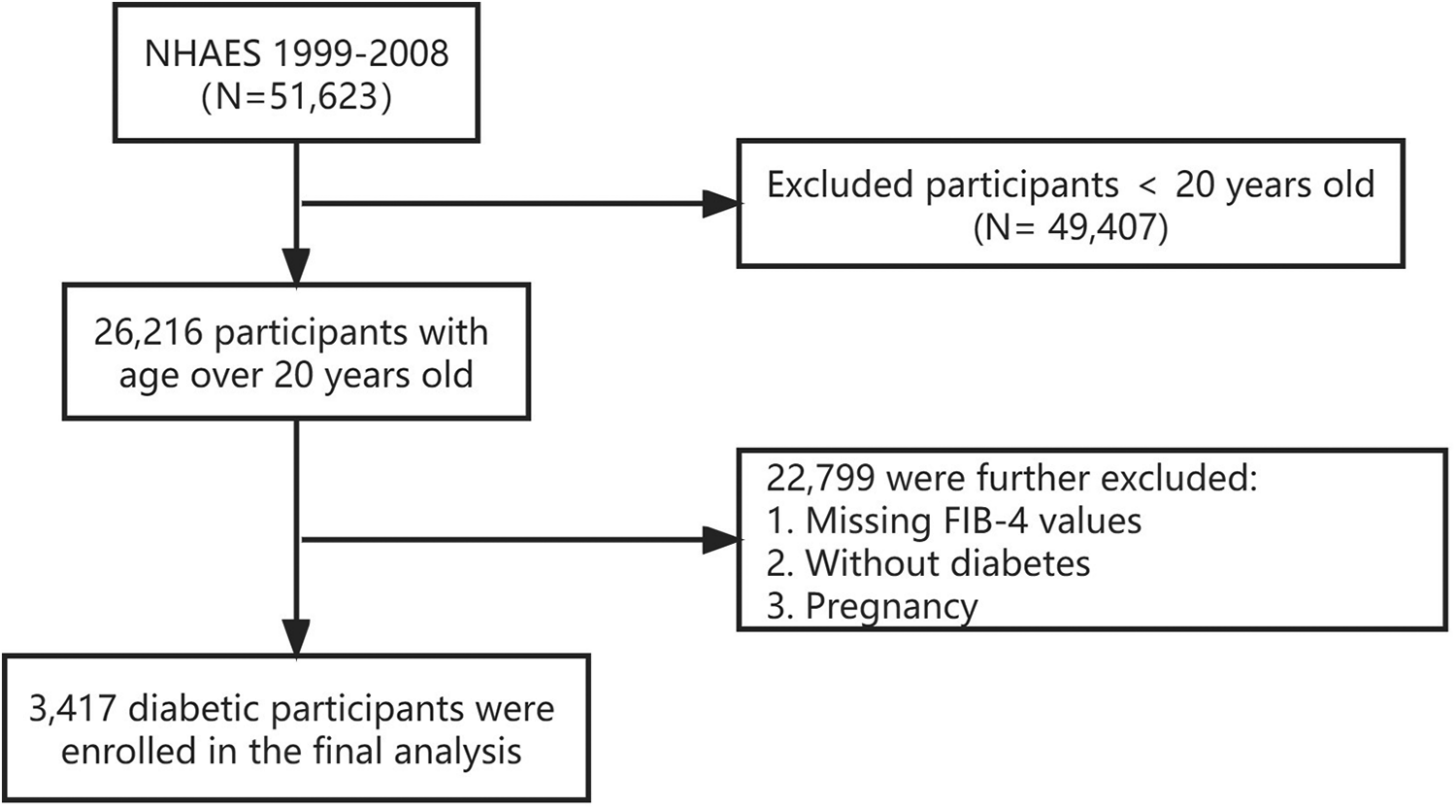
Flowchart of participants selection. NHANES, the National Health and Nutrition Examination Survey. FIB-4, fibrosis-4 index.

In the present study, we combined five NHANES cycles from 1999 to 2008 (*N* = 51,623) and obtained demographic, examination, laboratory, and questionnaire data from each cycle. Diabetes is defined in combination of the following criteria of the American Diabetes Association (14) and the questionnaire: (1) fasting glucose level ≥126 mg/dl, 2-h PG ≥200 mg/dl, or glycohemoglobin (HbA1c) ≥6.5%; (2) self-reported diabetes medical diagnosis; (3) taking antidiabetic drugs or insulin. Diabetes is determined by meeting one of the above criteria. Our study included 26,216 participants who were at least 20 years old. We excluded participants without diabetes, those missing FIB-4 results, or pregnant women (*N* = 22,799). Finally, 3,417 diabetic participants were enrolled in this study (Figure 1).

### 2.2. Data collection and laboratory methods

In the NHANES, sample collection follows a strict process and performed in the Mobile Examination Center (MEC) for standardization (15). Once the venous blood has been collected, it will be shipped to the central laboratory for testing. If samples are not tested within the specified time, they will be refrigerated or frozen at the appropriate temperature.

We collected data of serum aminotransferase (ALT), and aspartate aminotransferase (AST) activity, serum creatine and insulin concentration, blood HbA1c and platelet counts, and fasting plasma glucose from NHANES 1999 to 2008 database. Serum ALT, AST, and creatine were included in standard biochemistry profile and were measured through a biochemical analyzer. Serum ALT or AST activity was measured by α-ketoglutararate reaction. Serum creatine was measured through Jaffe reaction. Serum insulin was measued by a double-antibody radioimmunoassay. Fating plasma glucose was detemined by hexokinase reaction. Blood HbA1c was measured through boronate-affinity high performance liquid chromatography. Platlet count was performed on Beckman Coulter.

### 2.3. Calculation of serum FIB-4 concentrations

In this study, Fibrosis-4 index (FIB-4), an index related to liver fibrosis, was identified as exposure variable. FIB-4 was calculated by the following formula (16):

$$\mathrm{FIB}-4=(\left(\mathrm{Age}(\mathrm{Years})\times \mathrm{AST}(U/L)\right)/\left(\mathrm{platlet}\;\mathrm{conunts}(10^{9}/L)\times \mathrm{AL}T^{\frac{1}{2}}(U/L)\right)$$

### 2.4. Determination of outcomes

To obtain the mortality status of the follow-up population, we adopted the data from Linked Mortality Files collected by NCHS, which uses a probability match to link NHANES participant data with National Death Index death certificate data, which used the International Statistical Classification of Diseases to determine the cause of death.

Cardiovascular disease was defined as a self-reported medical diagnosis of coronary heart disease, stroke, angina pectoris, myocardial infarction, or congestive heart failure by a standard medical condition questionnaire (17).

### 2.5. Assessment of covariates

The demographic and lifestyle data in NHANES were obtained through questionnaires collection during home interviews; the data include age (age in years of participants at time of screening), sex, race/ethnicity, marital status, alcohol intake, smoking status, and disease status (history of hypertension, congestive heart failure, coronary heart disease, angina pectoris or myocardial infarction). The weight, height, body mass index (BMI, kg/m^2^), and waist circumference of the participants were measured at Mobile Examination Center.

Furthermore, race/ethnicity was classified as Mexican American, non-Hispanic white, non-Hispanic black, or other; marital status was classified as married, living with a partner, divorced, widowed, separated, or never married. As for smoking status, the participants were divided into former (smoked ≥100 cigarettes in life but did not smoke amid of survey), never smoke (smoked <100 cigarettes in life), and current smoker (smoked ≥100 cigarettes in life and smoked amid of survey) (18). Alcohol intake was classified into never, mild, moderate, and heavy based on the average drinks per week during the past 12 months (0, <1, 1−<8, and ≥8 drinks per week, a drink refers to a 12 oz. beer, a 4 oz. glass of wine, or an ounce of liquor) and former alcohol user (no alcohol intake amid of survey) (19). Furthermore, hypertension was defined as systolic blood pressure ≥40 mmHg or diastolic blood pressure ≥ 90mmHg (20), a self-reported hypertension medical diagnosis, or a self-reported history of taking blood pressure drugs.

The collection of serum specimens followed strict blood collection and analysis procedures. This study also reported the liver function indexes (AST, U/L; ALT, U/L), glucose regulation indexes (fasting glucose (mg/dl), insulin (pmol/L), and glycohemoglobin (%)), and platelet count (1,000 cells/ul). Furthermore, eGFR—an index of renal function—was calculated according to Chronic Kidney Disease Epidemiology Collaboration equation (21).

### 2.6. Statistical analysis

This study's data analysis followed the NHANES guideline (22). Continuous variables with a normal or approximately normal distribution are expressed as the mean with standard deviation, while the skewed distribution variables are represented as the median (interquartile, IQR). Categorical variables are reported as the number with a percentage. The *χ*^2^ test, one-way ANOVA, and Kruskal-Wallis test were used to compare the differences in categorical variables, continuous variables with normal distribution, and continuous variables with skewed distribution, respectively. The non-linear relationship between FIB-4 and all-cause or cardiovascular mortality was analyzed using restricted cubic splines. Restricted cubic splines were used 4 nodes and median as a reference point to examine the nonlinear association within the values to minimize the effect the potential outliers. The Cox regression models were used to investigate the influence of FIB-4 alteration on the hazard ratios (HRs) of all-cause and cardiovascular mortality. Model 1 adjusted for age and sex. Model 2 further included adjustments for race/ethnicity, marital status, alcohol drinking, and smoking habits. Model 3 added BMI, hypertension, family history of CVD, and eGFR. The logistic regression models were used to investigate the relationship between FIB-4 and CVD and its subtypes and results were expressed as odds ratios (ORs).

Furthermore, we conducted a stratified analysis of FIB-4 value based on age (<60 or ≥60 years), sex (male or female), BMI (<25 or ≥25 kg/m^2^), hypertension (yes or no), and eGFR (<90 ml/min/1.73 m^2^ or ≥90 ml/min/1.73 m^2^). To further investigate the association between FIB-4 and CVD, we adopted binary logistic regression models to analyze changes in odds ratios of CVD and CVD subtypes (congestive heart failure, coronary heart disease, angina pectoris, or myocardial infarction) for each SD increase in FIB-4.

The obtained data were analyzed and visualized using Empower stats 2.0 and statistical software R (version 3.4.3). *P* < 0.05 was considered statistically significant.

## 3. Result

### 3.1. Basic characteristics of the study population

Table 1 summarizes the basic characteristics of the participants. A total of 3,417 participants were included in our study, with an average age of 59.06 years, and 51.95% were male. The participants were divided into the survivor group (*n* = 1,785) and the mortality group (*n* = 1,632). Mortality people had higher FIB-4 values (1.65 ± 0.03) than the survivor people (1.07 ± 0.02). In the survivor group, the average age was 53.34 years, which was younger than in the mortality group (67.82 years). In the survivor group, there were 48.39% males and 60.93% non-Hispanic white people. Compared with the participants in the mortality group, those in the survivor group were more likely to be married, obese, heavy drinkers, and less likely to smoke. Besides, a lower proportion of the survivor group exhibited various CVDs (coronary heart disease, stroke, angina, heart attack, or congestive heart failure). Moreover, the survivor group exhibited higher ALT, AST, and eGFR, indicating improved liver and kidney function. However, the two groups had no difference in glucose regulation indexes (fasting glucose, insulin, and HbA1c).

**Table 1:** Basic characteristics of the participants.

Characteristics	Total	Survivor	Mortality	*P* value
*n* (%)	3,417	1,785 (51.43)	1,632 (47.02)	** **
Age, years	59.06 (0.40)	53.34 (0.39)	67.82 (0.42)	<0.0001
Sex, *n* (%)				0.058
Male	1,775 (51.95)	877 (48.39)	898 (52.80)	
Female	1,642 (48.05)	908 (51.61)	734 (47.20)	
Race/ethnicity, *n* (%)				<0.0001
Mexican American	824 (24.11)	513 (10.52)	311 (4.83)	
Non-Hispanic white	1,405 (41.12)	589 (60.93)	816 (72.89)	
Non-Hispanic black	845 (24.73)	449 (15.31)	396 (13.96)	
Other	343 (10.04)	234 (13.24)	109 (8.31)	
Marital status, *n* (%)				<0.0001
Married	1,918 (57.03)	1,109 (65.63)	809 (51.38)	
Living with a partner	99 (2.94)	73 (3.70)	26 (2.45)	
Separated	117 (3.48)	70 (2.94)	47 (2.14)	
Divorced	378 (11.24)	187 (10.85)	191 (13.80)	
Never married	257 (7.64)	164 (10.18)	93 (5.63)	
Widowed	594 (17.66)	167 (6.69)	427 (24.60)	
BMI, kg/m^2^	32.33 (0.23)	33.17 (0.28)	30.99 (0.30)	<0.0001
Waist circumference, cm	109.13 (0.54)	109.79 (0.66)	108.03 (0.72)	0.048
Alcohol intake, *n* (%)				<0.0001
Former	1,125 (35.12)	496 (26.41)	629 (40.10)	
Never	616 (19.23)	293 (16.33)	323 (20.38)	
Mild	837 (26.13)	479 (30.36)	358 (26.10)	
Moderate	269 (8.4)	176 (13.12)	93 (6.69)	
Heavy	356 (11.11)	238 (13.78)	118 (6.72)	
Smoking, *n* (%)				<0.0001
Never	1,607 (47.11)	921 (52.96)	686 (40.57)	
Former	1,211 (35.5)	541 (28.88)	670 (40.64)	
Current	593 (17.38)	319 (18.16)	274 (18.79)	
Hypertension, *n* (%)				<0.0001
Yes	2,530 (74.04)	1,196 (63.15)	1,334 (79.57)	
No	887 (25.96)	589 (36.85)	298 (20.43)	
Coronary heart disease, *n* (%)				<0.0001
Yes	367 (10.86)	102 (5.79)	265 (18.63)	
No	3,012 (89.14)	1,676 (94.21)	1,336 (81.37)	
Stroke, *n* (%)				<0.0001
Yes	318 (9.33)	86 (4.25)	232 (15.30)	
No	3,092 (90.67)	1,696 (95.75)	1,396 (84.70)	
Angina, *n* (%)				<0.0001
Yes	281 (8.33)	81 (3.66)	200 (13.89)	
No	3,092 (91.67)	1,692 (96.34)	1,400 (86.11)	
Heart attack, *n* (%)				<0.0001
Yes	395 (11.59)	100 (5.18)	295 (19.44)	
No	3,012 (88.41)	1,684 (94.82)	1,328 (80.56)	
Congestive heart failure, *n* (%)				<0.0001
Yes	323 (9.54)	67 (3.33)	256 (17.39)	
No	3,061 (90.46)	1,708 (96.67)	1,353 (82.61)	
Glucose, mg/dl	153.64 (2.10)	150.90 (2.82)	157.66 (2.87)	0.091
Insulin, pmol/L	120.20 (4.08)	114.83 (4.32)	128.07 (8.12)	0.162
HbA1c, %	7.09 (0.04)	7.10 (0.06)	7.07 (0.06)	0.678
ALT, U/L	28.02 (0.63)	29.78 (0.92)	25.32 (0.61)	<0.0001
AST, U/L	26.70 (0.49)	26.83 (0.71)	26.50 (0.49)	0.688
eGFR, ml/min/1.73 m^2^	83.03 (0.65)	91.01 (0.63)	70.82 (0.80)	<0.0001
FIB-4	1.30 (0.02)	1.07 (0.02)	1.65 (0.03)	<0.0001

### 3.2. FIB-4 levels and all-cause mortality

**Figure 2:**
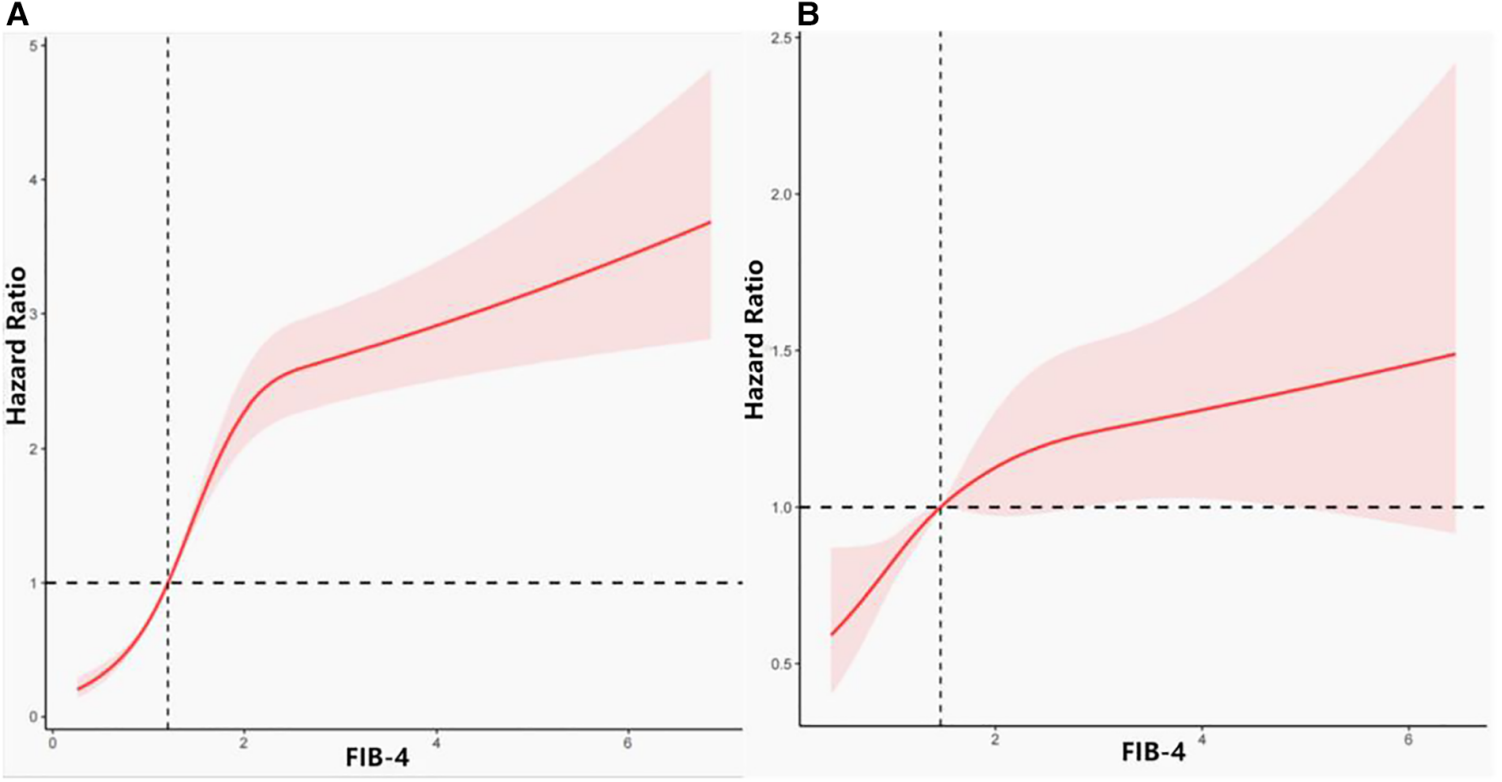
Association between FIB-4 and all-cause and cardiovascular disease mortality. (**A**) Association between FIB-4 and all-cause mortality. (**B**) Association between FIB-4 and cardiovascular mortality.

We analyzed the relationship between FIB-4 levels and all-cause mortality through restricted cubic splines based on the multivariable Cox proportional hazards model 3 (Figure 2A). The all-cause risk was increased sharply with an increase in FIB-4 initially and then steadily (*P* for non-linearity < 0.001). After multivariate adjustment including age, sex, race/ethnicity, marital status, alcohol drinking, smoking habits, BMI, hypertension, diabetes, and family history of CVD, eGFR, and HbA1c (Model 3), the trend of the hazards model did not differ from the unadjusted model, but the risk for each SD increase in FIB-4 decreased from 31% (HR 1.31; 95% CI, 1.20–1.44) to 24% (HR 1.24; 95% CI, 1.17–1.32) in the adjusted model (Table 2).

**Table 2:** Risk ratios of all-cause and cardiovascular mortality by FIB-4 value among diabetic participants in NHANES III (1999–2008).

Models	Hazard ratios (95% confidence interval)	*P*-value
All-cause mortality		
Unadjusted	1.31 (1.20, 1.44)	<0.0001
Model 1	1.19 (1.13, 1.25)	<0.0001
Model 2	1.11 (1.07, 1.15)	<0.0001
Model 3	1.24 (1.17, 1.32)	<0.0001
Cardiovascular mortality		
Unadjusted	1.16 (1.04, 1.31)	0.012
Model 1	1.17 (1.04, 1.31)	0.012
Model 2	1.16 (1.03, 1.30)	0.011
Model 3	1.17 (1.04, 1.31)	0.011

Model 1: adjusted for age and sex.

Model 2: further adjusted for race/ethnicity, marital status, alcohol drinking, and smoking habits.

Model 3: further adjusted for BMI, hypertension, family history of CVD, and eGFR.

### 3.3. FIB-4 levels and cardiovascular mortality

Figure 2B depicts the relationship between FIB-4 levels and cardiovascular mortality through restricted cubic splines based on Model 3. The overall trend of the model was similar to that of all-cause mortality. The risk of cardiovascular mortality increased with the increase in FIB-4 values. After adjusting for all possible confounders, the increased risk of cardiovascular mortality in relation with FIB-4 did not change significantly from 16% (unadjusted, HR 1.16; 95% CI, 1.04–1.31) to 17% (Model 3, HR 1.17; 95% CI, 1.04–1.31) (Table 2).

### 3.4. Relationship between FIB-4 levels and CVD and CVD subtypes

Table 3 presents the unadjusted and adjusted relationship model between FIB-4 values and CVD and CVD subtypes. When adjusting for all covariates, elevated FIB-4 values were associated with CVD (OR 1.15; 95% CI, 1.02–1.31). Furthermore, we analyzed the relationship between FIB-4 and four CVD subtypes (congestive heart failure, coronary heart disease, angina pectoris, and myocardial infarction). As shown in Table 3, after adjusting for all covariates, FIB-4 values were associated with myocardial infarction (OR 1.25; 95% CI, 1.05–1.47). However, there was no significant relationship between FIB-4 and the other three CVD subtypes (congestive heart failure, coronary heart disease, and angina pectoris) (Supplementary Figure S1). Since the participants included were those present with CVD history, we were unable to investigate the prospective relationship between FIB-4 and CVD subtypes.

**Table 3:** Odds ratios of CVD and CVD subtypes risk by FIB-4 value among diabetic participants in NHANES III (1999–2008).

Models	Odds ratios (95% confidence interval)	*P*-value
CVD		
Unadjusted	1.73 (1.49, 2.00)	<0.0001
Model 1	1.12 (1.00, 1.26)	0.048
Model 2	1.13 (1.00, 1.27)	0.041
Model 3	1.15 (1.02, 1.31)	0.032
Congestive heart failure		
Unadjusted	1.40 (1.24, 1.57)	<0.0001
Model 1	1.08 (0.92, 1.26)	0.333
Model 2	1.08 (0.93, 1.26)	0.313
Model 3	1.08 (0.90, 1.29)	0.402
Coronary heart disease		
Unadjusted	1.46 (1.26, 1.67)	<0.0001
Model 1	1.06 (0.91, 1.24)	0.421
Model 2	1.06 (0.91, 1.24)	0.432
Model 3	1.07 (0.92, 1.26)	0.377
Angina pectoris		
Unadjusted	1.28 (1.12, 1.46)	<0.001
Model 1	1.00 (0.76, 1.31)	0.999
Model 2	1.00 (0.76, 1.31)	0.891
Model 3	1.02 (0.76, 1.36)	0.901
Myocardial infarction		
Unadjusted	1.56 (1.35, 1.81)	<0.0001
Model 1	1.22 (1.05, 1.41)	0.011
Model 2	1.23 (1.04, 1.41)	0.010
Model 3	1.25 (1.05, 1.47)	0.011

Model 1: adjusted for age and sex.

Model 2: further adjusted for race/ethnicity, marital status, alcohol drinking, and smoking habits.

Model 3: further adjusted for BMI, hypertension, family history of CVD, and eGFR.

### 3.5. Stratified analysis

**Figure 3:**
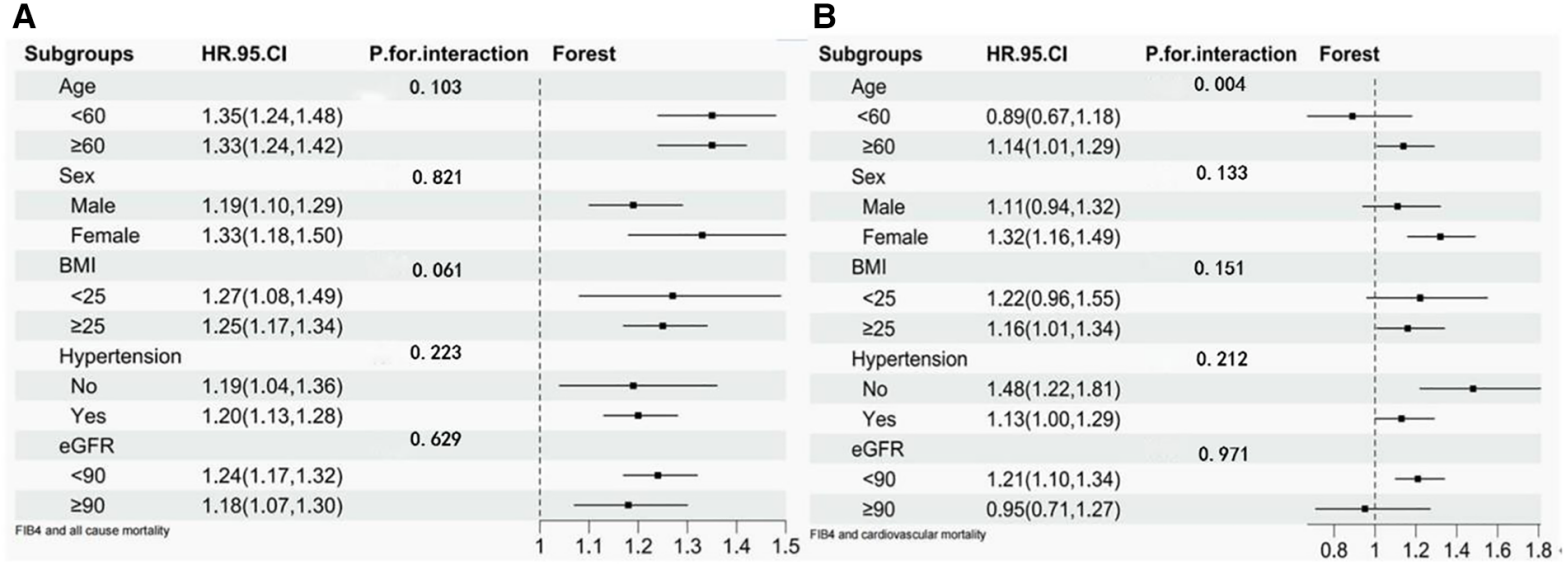
Stratified analysis of FIB-4 and risk of all-cause mortality and cardiovascular mortality. (**A**) Stratified analysis of FIB-4 and risk of all-cause mortality. (**B**) Stratified analysis of FIB-4 and risk of cardiovascular mortality.

In stratified analysis, participants were divided into a wide range of subgroups stratified by age, sex, BMI, hypertension, or eGFR. As shown in Figure 3, stratified variables had no significant effect on the relationship between FIB-4 and all-cause mortality. Regarding the cardiovascular mortality risk, the relationship between FIB-4 and CVD was not influenced by sex, BMI, hypertension, and eGFR. However, FIB-4 had a considerable influence on cardiovascular mortality among participants older than 60 years. Each SD increment in FIB-4 increased the risk of CVD mortality by 14% (HR 1.14; 95% CI 1.01–1.29).

## 4. Discussion

The present study adopted nationwide, cross-sectional data of the US diabetic population from NHANES (1999–2008) to explore the relationship between FIB-4 and CVD. Higher FIB-4 values increased the risk of all-cause and CVD mortality. Furthermore, we found that FIB-4 was positively associated with the incidence of CVD and its subtype, myocardial infarction, but not associated with the other three subtypes. In stratified analysis, other covariates, except for age, did not affect the association between FIB-4 and CVD mortality. FIB-4 increased the mortality risk of CVD in the participants older than 60 years, but it did not affect the participants younger than 60 years.

FIB-4 is a highly-sensitive biomarker for evaluating advanced liver fibrosis. However, in recent years, studies have begun to focus on the predictive value of FIB-4 on the occurrence and prognosis of CVD (23–25), indicating the importance of liver fibrosis in developing CVD. In our study, we found that mortality diabetic participants had higher FIB-4 values and that increasing FIB-4 was associated with an increased risk of CVD incidence and mortality, consistent with the results of the previous study. A prospective survey of heart failure with preserved ejection fraction (HFpEF) patients suggested that FIB-4 ≥3.11 before discharge had a 2.202-fold increased risk of major adverse cardiovascular events (MACE), which was defined as death, readmission for heart failure, nonfatal myocardial infarction, and nonfatal stroke (26).

Considering the importance of FIB-4 in predicting liver fibrosis, the mechanisms of FIB-4 in predicting CVD may be associated with the relationship between liver fibrosis and CVD. Liver fibrosis—resulting from abnormal liver fat metabolism—may cause abnormal accumulation of liver fat, leading to inflammation, glucose metabolism disorder, and eventually an increase in CVD incidence (25, 27). A previous study found that the histological severity of liver fibrosis can be an independent risk factor in predicting the incidence of CVD (28), indicating the potential role of FIB-4 in indirectly predicting CVD. However, a multicenter study in Sweden revealed that the degree of liver fibrosis did not independently affect the risk of CVD (29).

In our study, we found that CVD subtypes, except myocardial infarction, were not associated with FIB-4 after excluding all possible confounders, which contradicts the findings of some previous studies (30–32). The possible reasons may be as follows. First, FIB-4 may not be an ideal biomarker to identify the relationship between liver fibrosis and CVD. A Chinese cohort study on HFpEF patients revealed that although advanced liver fibrosis increased the incidence of new-onset atrial fibrillation (AF), NAFLD fibrosis score, another non-invasive liver biomarker, was associated with increased AF incidence rather than FIB-4 (33). Furthermore, some research has indicated a limitation of FIB-4 in predicting liver fibrosis among diabetic patients (34, 35), suggesting that FIB-4 may not play a role in demonstrating the relationship between liver fibrosis and CVD in diabetic patients. Second, as FIB-4 performance varies owing to the prevalence across various populations, the cut-off value for FIB-4 must be adjusted based on the sample population, known as the “spectrum effect” (36, 37), so the prediction value of FIB-4 may be affected by the cut-off value. A Japanese study on AF patients revealed that FIB-4 ≥2.51 was independently associated with CVD events and all-cause mortality (31). Nevertheless, another study found that a high probability of liver fibrosis set by FIB-4 ≥1.30 was not independently associated with atherosclerosis, the crucial cause of CVD (38). Besides, a previous study suggested that the optimized cut-off value of FIB-4 in predicting liver cirrhosis among the diabetic population was 2.96 (39). In the present study, however, in the dead group or the survivor group, the mean FIB-4 was lower than 2.96, indicating that the participants in our study may not exhibit significant liver fibrosis. However, the optimized cut-off of 2.96 may not be suitable for the present study because of the different sample populations. Thus, the relationship between liver fibrosis represented by FIB-4 and CVD subtypes should be further investigated.

To further investigate the effects of possible covariates on the relationship between FIB-4 and CVD, we conducted a stratified analysis based on age, sex, BMI, hypertension, and eGFR. We found that FIB-4 values had different effects on CVD mortality in participants older than 60 years. Our study revealed that higher FIB-4 might increase the death of CVD in diabetic patients older than 60 years, whereas FIB-4 had no effect on CVD mortality among participants younger than 60 years old. This finding may be partly attributed to age, including in the FIB-4 equation. Moreover, a recent study on Korean people older than 50 years revealed that higher FIB-4 could predict all-cause or CVD mortality (24), which is consistent with the findings of our study. Another study revealed that adopting age-specific cut-off values exhibited slightly lower HR of CVD (HR 1.60; 95% CI, 1.27–2.01) than alternative cut-offs (FIB ≥3.25, HR 1.94; 95% CI, 1.37–2.73; FIB ≥2.67, HR 1.81; 95% CI, 1.43–2.29) in previous studies (25). Furthermore, a previous study revealed the importance of establishing an age-specific reference interval of FIB-4, especially among people older than 65 years, which can increase the accuracy of its prediction of liver fibrosis (40) and may help strengthen the relationship between FIB-4 and CVD. As for hypertension and BMI, which are clinical risk factors of CVD, we showed that they did not affect the association between FIB-4 and CVD risk, which was consistent with previous study (25). Moreover, diabetic kidney disease is one of the most frequent complications of diabetes and also strongly associated with CVD. Thus, it is important to exclude kidney disease from interfering with the relationship between FIB-4 and CVD (41). eGFR is a biomarker reflecting the impairment of renal function and was used in this study to investigate the influence of kidney disease. However, we found eGFR did not affect the relationship between FIB-4 and CVD. Accordingly, dividing participants into subgroups based on age allows for a better understanding of the effect of FIB on CVD.

The strengths of our study include national-based data, a large sample size, and detailed sample information, reflecting the representative and robustness of our study. However, our study has some limitations. First, NHANES is a cross-section survey of the US population that only recorded the baseline information at the time of inclusion. Thus, we could not elucidate the casual association between FIB-4 and CVD and identify potential covariable changes over time. Second, there may be memory bias, misreporting, and misrepresentation, although some data, such as disease status, smoking condition, and alcohol intake, were self-reported through questionnaires. Third, though our study did not include other possible covariates, such as physical activity and dietary habits, which may affect the results. Therefore, it is worthwhile to stratify FIB-4 based on different cut-offs to investigate the relationship between FIB-4 and CVD.

## 5. Conclusion

In conclusion, we adopted NHANES (1999–2008) data to assess the relationship between FIB-4 and all-cause and CVD mortality in the US diabetic population. FIB-4 was strongly associated with all-cause and CVD mortality, reflecting that liver fibrosis may result in higher mortality among diabetic people. Nevertheless, FIB-4 was associated only with the incidence of myocardial infarction but not with other CVD subtypes. Furthermore, FIB-4 was deeply influenced by age, which increased the risk of CVD mortality in participants older than 60 years. Future research should focus on the predictive role of FIB-4 in CVD mortality among diabetic people, especially older people. Furthermore, more in-depth research should be conducted on the effect of FIB-4 on the incidence risk of CVD and its subtypes.

## Data Availability

The original contributions presented in the study are included in the article/**Supplementary Material**, further inquiries can be directed to the corresponding author.
