# Supplementary material for: Association between FIB-4, all-cause mortality, cardiovascular mortality, and cardiovascular disease risk among diabetic individuals: NHANES 1999–2008
Source: Front Cardiovasc Med. 2023 Sep 25;10:1172178. doi: 10.3389/fcvm.2023.1172178 (PMC10560879; doi:10.3389/fcvm.2023.1172178)
Supplement: Supplementary file 1 [file Table1.docx]

Supplementary Material

Association between FIB-4, all-cause mortality, cardiovascular mortality, and cardiovascular disease risk among diabetic individuals: NHANES 1999-2008

**Lihua Guan^1†^, Lei Li^1†^, Yutong Zou^1^, Jian Zhong^1^, Ling Qiu^1,2^**

*** Correspondence:** Ling Qiu: lingqiubj@163.com


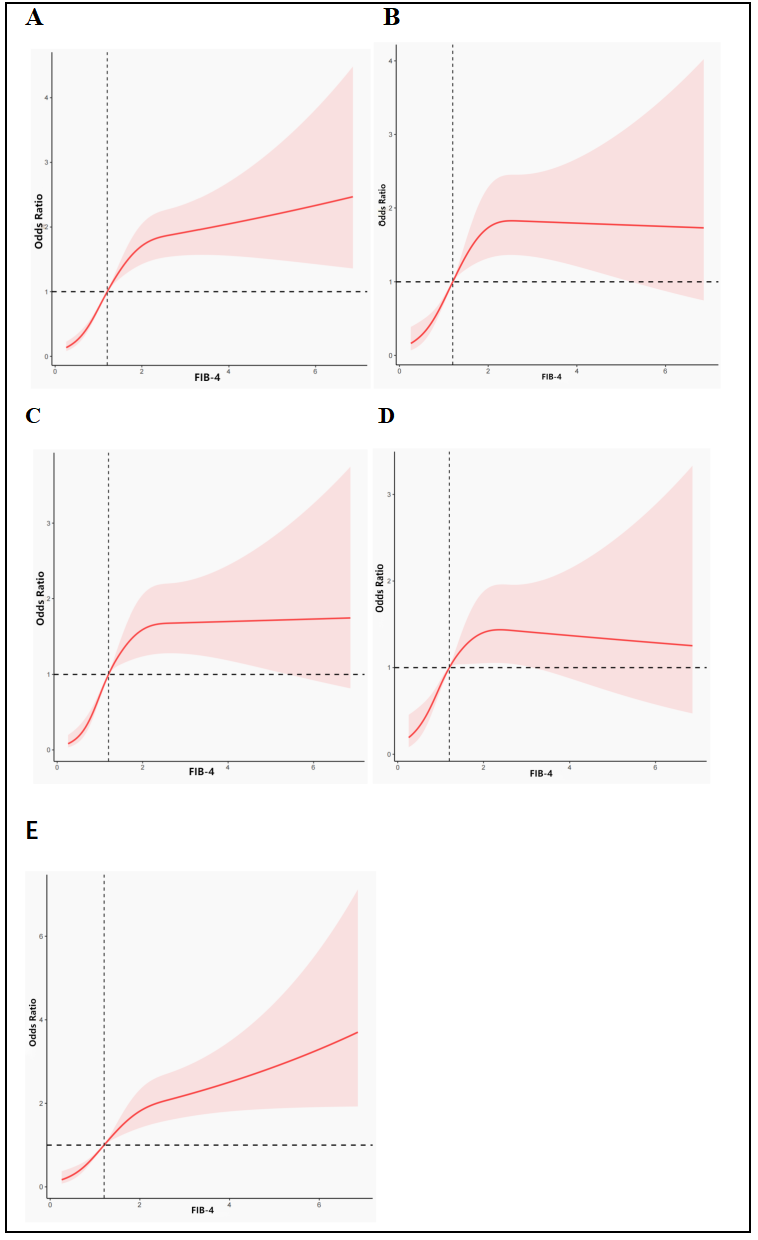


**Supplementary Figure 1.** **Association between FIB-4 and risk of cardiovascular disease and its subtypes.**

1. Association between FIB-4 and risk of CVD.
2. Association between FIB-4 and congestive heart failure.
3. Association between FIB-4 and coronary heart disease.
4. Association between FIB-4 and angina pectoris.
5. Association between FIB-4 and Myocardial infarction.
